# Classification of rat mammary carcinoma with large scale in vivo microwave measurements

**DOI:** 10.1038/s41598-021-03884-7

**Published:** 2022-01-10

**Authors:** Emre Onemli, Sulayman Joof, Cemanur Aydinalp, Nural Pastacı Özsobacı, Fatma Ateş Alkan, Nuray Kepil, Islem Rekik, Ibrahim Akduman, Tuba Yilmaz

**Affiliations:** 1 Department of Electronics and Communication Engineering, Istanbul Technical University, Istanbul, 34469 Turkey; 2 Mitos Medical Technologies, ITU Ayazaga Ari Teknokent 2-B Block 2-2-E, Maslak Istanbul, 34469 Turkey; 3 Department of Biophysics, Cerrahpasa Medical School, Istanbul University-Cerrahpasa, Istanbul, 34098 Turkey; 4 Department of Biophysics, Medical School, Beykent University, Istanbul, 34520 Turkey; 5 Department of Pathology, Cerrahpasa Medical School, Istanbul University-Cerrahpasa, Istanbul, 34098 Turkey; 6 Faculty of Computer and Informatics Engineering, Istanbul Technical University, Istanbul, 34469 Turkey

**Keywords:** Biomedical engineering, Breast cancer, Electrical and electronic engineering

## Abstract

Mammary carcinoma, breast cancer, is the most commonly diagnosed cancer type among women. Therefore, potential new technologies for the diagnosis and treatment of the disease are being investigated. One promising technique is microwave applications designed to exploit the inherent dielectric property discrepancy between the malignant and normal tissues. In theory, the anomalies can be characterized by simply measuring the dielectric properties. However, the current measurement technique is error-prone and a single measurement is not accurate enough to detect anomalies with high confidence. This work proposes to classify the rat mammary carcinoma, based on collected large-scale in vivo S$_{11}$ measurements and corresponding tissue dielectric properties with a circular diffraction antenna. The tissues were classified with high accuracy in a reproducible way by leveraging a learning-based linear classifier. Moreover, the most discriminative S$_{11}$ measurement was identified, and to our surprise, using the discriminative measurement along with a linear classifier an 86.92% accuracy was achieved. These findings suggest that a narrow band microwave circuitry can support the antenna enabling a low-cost automated microwave diagnostic system.

## Introduction

Inherent dielectric property discrepancy between the malignant breast tumors and healthy breast tissues at microwave frequencies^1,2^ has fueled the research in development of microwave imaging and hyperthermia applications^3,4^, while potential other use of microwaves, such as microwave surgical margin detection and microwave biopsy, remains overlooked. Currently, both diagnostics applications are based on pathology and performed by specialists. Thus, these processes are costly and prone to human error, particularly biopsy suffers from low success rates for detection of ductal breast carcinoma^5^.

Circular diffraction antenna^6^, commonly referred as the open-ended coaxial probe, can be utilized for both pathological applications either as a complimentary or sole diagnosis technique. In theory, this can be realized simply by measuring the dielectric property discrepancy between healthy and malignant tissues. Ultimately, the practical applications, such as microwave biopsy probe, of open-ended coaxial probes was previously envisioned in the literature^7^. However, the proposed applications was not followed with the necessary research and development to realize the potential practical applications of open-ended coaxial probes. Inherently high error rates are the primary limitation of the technique preventing the transition from laboratory to the clinical practice. Therefore, open-ended coaxial probes are currently only commercially available for laboratory use and mostly utilized for dielectric property characterization to enable the development of many other microwave based applications. Commercially available kits reports a measurement error of 5% for laboratory use, while it is reported in the literature that the measurement error can be as high as 30% for an in vivo practical measurement setting^8^.

There are a number of error sources contributing the high measurement error rates which can be categorized as system, user, and sample related errors. System related errors include mathematical approach, calibration and probe deterioration. Particularly, dielectric properties are not directly measurable quantities; thus, derived from other measurable quantities namely S-parameters. For open-ended coaxial probes S$_{11}$ response is used; therefore, S-parameter refers to S$_{11}$ response through this paper. Each mathematical approach has its own limitations including but not limited to inability to converge and narrow band applicability^9^. The user related errors include cable movements, applied pressure, and inconsistent calibration or measurement practices^10^. Yet another error source is sample related measurements including changing sample temperature and sample heterogeneity^11^. These error sources are usually managed by collecting multiple measurements from a sample in a laboratory setting following the best measurement practices. However, to realize a microwave surgical margin detection or microwave biopsy device, single and high accuracy measurements must be collected from the biological tissues in an in vivo clinical setting. Therefore, there is a need to address the error sources to enable the microwave diagnostic and therapeutic applications of the open-ended coaxial probes.

Dielectric properties of the breast carcinoma were investigated in several studies. Cho et al. measured the xenografted breast carcinoma tissues on nude mice using an insertion-type planar probe^12^. The study reported 97$\%$ accuracy; however, the invasive characteristic of this probe type is not suitable for surgical margin detection. Additionally, this work did not integrate automated decision making mechanisms. Kim and Pack designed a sample holder placed between two ports^13^. The reported study is not suitable for in vivo tissue characterization., Martellosio et al. measured dielectric properties of breast tissue samples obtained from breast surgeries using open-ended coaxial probe technique. Reported study is limited to ex vivo measurements and 80$\%$ classification accuracy^14^. A similar study was conducted by Cheng and Fu on ex vivo human breast tissues^15^. Yet, the reported work is limited to statistical analysis. Although the reported studies contribute to the dielectric property knowledge of breast tissues, both in vivo dielectric properties and S-parameter reponses have not been analyzed extensively in the literature.

To this end, this work investigates (1) the effectiveness of linear classifiers to categorize the in vivo dielectric property data collected from the malignant and healthy rat mammary tissues, (2) the possibility of utilizing S-parameter response of open-ended coaxial probe for rat mammary tissue categorization with linear classifiers, (3) identifying the most significant S-parameter response with respect to frequency using feature selection, (4) tissue categorization with the most discriminative S-parameter response using a linear classifier. Towards these goals, dielectric property and S$_{11}$ response of open-ended coaxial probe was measured with in vivo healthy and chemically induced malignant rat mammary tissues from 0.5 to 6 GHz. Briefly, S$_{11}$ response is a measure of power delivered to the radiating element, in this work the probe. It is known that the S$_{11}$ response is partly dependent on the dielectric properties of the environment terminating the probe’s open end. Collected S$_{11}$ responses from healthy and malignant tissues were classified with Support Vector Machines (SVM) algorithm. Next, S$_{11}$ responses were used to perform a feature selection (FS) algorithm to select the best features discerning two classes namely malignant and healthy. To the best of authors’ knowledge, this is the first study presenting large scale in vivo rat mammary tissue dielectric properties, the corresponding S-parameter responses, their classification results and lastly classification with a real S-parameter response at a single frequency. The most significant contributions of this work are two folds:

- we show that tissue classification can be performed by only using S$_{11}$ response,
- a single frequency measurement is adequate for accurate classification.

These contributions suggest that a low-cost microwave surgical margin detection or microwave biopsy device that is able to perform accurate autonomous detection of malignant breast tissues can be developed. Remainder of this paper is organized as follows: the results are given in “Results” section, discussions are given in “Discussion” section, the experimental design including measurement sample preparation and experimental setup is presented in “Animals” section and “Experimental setup” section, measurement steps are given in “In vivo measurements” section, pathological analysis criteria are presented in “Pathological analysis” section and lastly data processing approaches are given in “Data processing” section.

## Results

This section reports the dielectric properties and corresponding S-parameters collected from the in vivo rat mammary tissues using open-ended coaxial probes along with the classification results of these quantities. Measurements were performed between 0.5 GHz and 6 GHz, where the optimal trade-off is acquired between tissue attenuation and spatial resolution particularly in microwave imaging of biological tissues^16^. Next, the obtained tissue dielectric property data were classified using the SVM method. The performance of the classification model for different data combinations were compared. Classification was also performed using the S-parameter responses. Also, performance of the classification algorithm was investigated at narrow band frequencies using the feature selection method on S-parameter data.

### In vivo microwave dielectric properties

**Figure 1:**
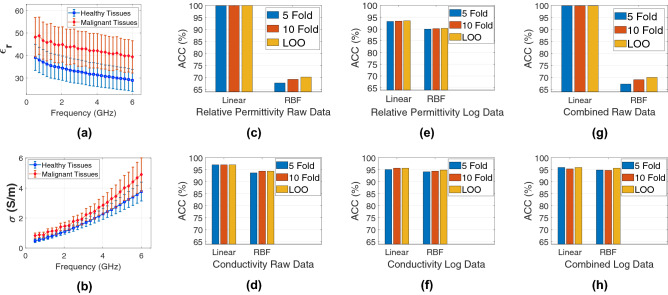
Classification results of the rat mammary carcinoma using three datasets derived from the microwave in vivo dielectric properties from 0.5 to 6 GHz. (**a**) Measured mean relative permittivities along with standard deviation from the mean (std) of healthy and malignant rat mammary tissues. (**b**) Measured mean conductivities along with std of healthy and malignant rat mammary tissues. (**c**) Classification accuracy (ACC) comparison of the Support Vector Machines (SVM) algorithm with linear and Radial Basis Function (RBF) kernels applied to raw relative permittivity data using different cross-validation (CV) schemes. (**d**) ACC comparison of the SVM algorithm with linear and RBF kernels applied to raw conductivity data using different CV schemes. (**e**) ACC comparison of the SVM with linear and RBF kernels applied to logarithm of the relative permittivity data using different CV schemes. (**f**) ACC comparison of the SVM algorithm with linear and RBF kernels applied to logarithm of the raw conductivity data using different CV schemes. (**g**) ACC comparison of the SVM with linear and RBF kernels applied to raw complex relative permittivity (combined) data using different CV schemes. (**h**) ACC comparison of the SVM with linear and RBF kernels applied to logarithm of the complex relative permittivity (combined) data using different CV schemes.

Dielectric properties were collected from healthy rat breast tissues of 5 animals and malignant rat breast tumors of 5 animals. A total of 650 measurements with 325 measurements from each group were utilized for the following analysis. Means of dielectric property measurement values obtained from healthy and malignant tissues along with the error bar denoting the standard deviation are shown in Fig. 1a,b for relative permittivity and conductivity, respectively. The difference between mean relative permittivity measurements of healthy and malignant rat breast tissues are 8.98 and 10.43 at 0.5 and 6 GHz, respectively. Difference between mean conductivity measurements of malignant and healthy tissues are 0.343 and 1.136 at 0.5 and 6 GHz, respectively. Two-way analysis of variance (ANOVA) method was implemented on collected dielectric property data for malignant and healthy rat breast tissues with 0.05 ($\alpha$) significance level. Statistically significant differences were found between two groups since p-value $\le$ $\alpha$ where p-value stands for the probability value.

Collected dielectric property data are separated into three dataset: (1) real part of relative permittivity ($\varepsilon _r$) with 101 features, (2) conductivity ($\sigma$ (S/m)) with 101 features, (3) complex relative permittivity ($\varepsilon ^*$=$\varepsilon _r$+j$\varepsilon {''}$) also referred as combined data with 202 features. The real part of permittivity represents the ability of the material to store the electromagnetic energy and imaginary part represents the loss of the material. Conductivity ($\sigma$) is obtained simply by multiplying the imaginary part with angular frequency (*omega* (rad/s)) and permittivity of free space ($\varepsilon _0$ (F/m)) ($\sigma$=$\omega \varepsilon _0 \varepsilon {''}$ (S/m)).

### Classification with in vivo dielectric property measurements

SVM algorithm was used for classification of all three dielectric property data. To evaluate the performances of learning algorithms different data pre-processing methods were used. We also propose to use multiple cross-validation (CV) schemes, including 5-fold, 10-fold, and leave-one-out (LOO) CV, to ensure the reproducibility of the obtained results. Raw and logarithm of the data were used independently while utilizing linear and Radial Basis Function (RBF) kernels. Note that the logarithm of the data was used as a pre-processing step. Performance was evaluated via implementing alternative combinations of (5-fold linear, 10-fold linear, LOO linear, 5-fold RBF, 10-fold RBF and LOO RBF) CVs with two kernel functions.

Comparisons of the obtained accuracy results for raw data are shown in Fig. 1c,d. Furthermore, the accuracy of pre-processed logarithm data was compared as shown in Fig. 1e,f. Finally, combined data performances were analyzed: relative permittivity and conductivity for raw data and logarithm data as shown in Fig. 1g,h, respectively. It can be clearly seen that linear SVM outperformed to RBF. Moreover, no significant difference was observed between CV schemes. The consistency of the accuracies obtained from different CV schemes ensures that the model is not under or overfitting the data. 10-fold and LOO have slightly better performance compared to 5-fold CV. Best results with 100% Accuracy obtained by using a combination of raw relative permittivity data with SVM linear kernel and raw complex relative permittivity data with SVM linear kernel. From these results it can be concluded that relative permittivity data is more discriminative than the conductivity data when used with a linear kernel. It should also be noted that the classifier gives reproducible results for all CV schemes. In the conductivity data linear and RBF kernels gives consistently close results. However, in raw relative permittivity and complex relative permittivity data the linear kernel outperforms the RBF kernel. With logarithmic pre-processing of the relative permittivity data the linear kernel accuracy decreases and RBF kernel accuracy increases, which indicates that the raw relative permittivity data is linearly separated.

### In vivo S-parameter responses

S$_{11}$ response represents the return loss at the aperture of the probe. The term S-parameter and S$_{11}$ are used interchangeably in this paper. S$_{11}$ based classification targets elimination of the data processing provided by a commercially available dielectric property measurement software. S-parameters were also used to investigate the accuracy of narrow band data through feature selection.

**Figure 2:**
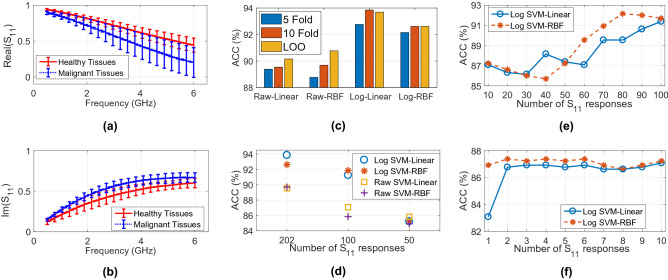
Classification pipeline of the rat mammary carcinoma, from data acquisition to analysis. (**a**) Real part of the antenna S$_{11}$ response when terminated with in vivo rat mammary tissues. (**b**) Imaginary part of the antenna S$_{11}$ response when terminated with in vivo rat mammary tissues. (**c**) Accuracy (ACC) comparison of the Support Vector Machines (SVM) with linear and Radial Basis Function (RBF) kernels applied to raw and logarithm of the raw data (S$_{11}$ response) using different cross-validation (CV) schemes. (**d**) ACC comparison for all 202, first 100, and first 50 S$_{11}$ response (features) for raw and logarithm of raw data with SVM algorithms with linear and RBF kernels. (**e**) ACC comparison of SVM with linear and RBF kernel for top scored S$_{11}$ response (feature) numbers varying from 10 to 100. (**f**) ACC comparison of SVM with linear and RBF kernel for top scored S$_{11}$ response (feature) numbers varying from 1 to 10.

Difference of the absolute mean S$_{11}$ responses between malignant and healthy tissues is shown in Fig. 2a,b. The differences between mean S$_{11}$ of the malignant and healthy tissues were calculated as 0.05 and 0.24 for the real part at 0.5 and 6 GHz, respectively. Similarly, 0.03 and 0.07 were found for the imaginary part at 0.5 and 6 GHz, respectively. Two-way ANOVA was applied to the mean of the collected absolute S$_{11}$ response for malignant and healthy tissues with 0.05 ($\alpha$) significance level. Statistically significant differences were found between two groups with p value $\le$ $\alpha$.

### Classification based on S-parameter responses

The raw S$_{11}$ response is a complex number where the real and imaginary parts of the responses were treated as different features in the machine learning algorithm. Since there was 101 measurement points between 0.5 to 6 GHz, the total number of features for each measurement sample was 202.

For classification the pre-processing and classification steps presented in “Methods” section is applied to the complex S$_{11}$ response. In the first step, the raw data were processed with SVM algorithm to find the optimal kernel and CV scheme. Both the raw data and the logarithm of the data were tested with different combinations of linear, RBF kernels and 5-fold, 10-fold, LOO CV. Different CV schemes were again utilized for reproducibility purposes^17,18^. The best performance was obtained with a combination of the logarithm of the data and linear kernel with 93.85% accuracy. This is consistent with the in vivo dielectric property results where the best results again was obtained with linear kernel. No significant difference was observed between the CV schemes ensuring reproducibility, shown in Fig. 2c.

### Feature selection applied to the S-parameter responses

Since the implemented CV schemes consistently showed similar performances, 10-fold CV were used for feature selection (FS) to minimize the processing times. Nested CV algorithm was applied to optimize the $\alpha$ parameter of Inf-FS method as well as to separate the test set during parameter optimization to avoid overfitting. FS is performed in three steps; first, by ranking and scoring 100 and 50 most significant features for both the raw and logarithm of the raw data, given in Fig. 2d. The accuracy of SVM-linear and SVM-RBF algorithms were evaluated for all data groups. The logarithm of the data outperformed for all features (202) and for the first 100 features. Therefore, the logarithm of the raw data was used for the rest of the data analysis. Next, first 10 to 100 features were used to evaluate the accuracy of SVM algorithm with linear and RBF kernels, shown in Fig. 2e. The accuracy dropped from 91.63% to 87.23% with the decreasing number of features. Lastly, first 1 to 10 features were used with SVM algorithm with linear and RBF kernels, shown in Fig. 2f. It resulted in 86.92% accuracy using a single feature. During the feature ranking the features were identified and tracked, the first ranked feature corresponded to the real part of S$_{11}$ response at 610 MHz.

## Discussion

Recent works^19,20^ implemented the machine learning algorithms to different dielectric property data with over 98% accuracy. Linear classification algorithms showed consistently well classification accuracies for dielectric property data. To the best of our knowledge, this work represents classification accuracies obtained for the largest microwave in vivo dielectric property data obtained from the rat mammary tissues. It is shown in this work that the classification accuracy for the in vivo dielectric property data can be as high as 100%.

Another goal of this work is to completely eliminate the data processing part applied to the S$_{11}$ response of the open-ended coaxial probe to obtain the dielectric properties. This was done by using the S$_{11}$ response instead of dielectric properties. Findings indicate that the S$_{11}$ based classification is able to reach over 93% accuracy, 95.39% sensitivity, and 92.31% specificity using a crude SVM model. This approach eliminates the software requirements to obtain the dielectric properties and indicates that S-parameter based classification with high accuracy is possible. Further, this approach allowed exploration of the classification using a single frequency with feature selection. It is shown that a single frequency response can be used with 6% decrease in accuracy. The obtained sensitivity and specificity were 78.46% and 95.39%, respectively. A narrow band measurement enables low-cost microwave circuitry that decreases the cost of the end product significantly.

These results indicate that a single frequency measurement carries a large majority of the information, suggesting an end product operating at a single frequency can potentially achieve high accuracy classification. Moreover, single frequency operation can enable a device utilizing a narrow band microwave circuit operating at relatively low frequencies, 610 MHz S$_{11}$ response,which in turn aid producing a cost effective device that can be deployed to remote clinics. Classification accuracy can be further improved by either increasing the sample size or potentially tuning the existing model.

Further, in experiment group mammary carcinoma measurements were collected from grade I tumors; however, the tumors were at different stages. The tumor growth period from the first diagnosis to sacrifice varied between 11 days to 105 days. From the experiments it was observed that the tumors at early stages were rather more homogeneous and measured S$_{11}$ were more consistent. Contrarily, necrosis and fat tissues was observed at late stage tumors and S$_{11}$ response also varied with sample heterogeneity. In control group, the animals had homogeneous tissues. Therefore, the tissue heterogeneity was minimal comparing to experiment group. This can also be observed from the S$_{11}$ response given in Fig. 2a,b. This suggests that S$_{11}$ measurement response as well as the age of the animal and the time elapsed from diagnosis to treatment can be used as inputs to the machine learning algorithms; hence, potentially improve the automated diagnosis accuracy.

Open-ended coaxial probe method was commonly used for broadband dielectric property characterization in laboratory environment. Potential application of the existing open-ended coaxial probes in the field of microwave diagnostics was first introduced about a decade ago; however, the research on realization of such device have been stagnated due to the inherent high error rates of the technique. This manuscript explores a machine learning based approach to exploit the potential utilization of open-ended coaxial probes as a low cost and high accuracy automated diagnostic technology. This device can potentially aid physicians in diagnosing breast cancer tissues in real time which can lead to acceleration of the current medical diagnosis and treatment protocols followed during biopsy as well as cancer resection surgeries.

Our findings indicate that it is possible to increase the tissue characterization accuracy of the existing technique by approximately 30% and 23% for in vivo dielectric property and S-parameter data through adoption of machine learning algorithms without additional costly hardware improvements. To our surprise, the adoption of feature selection algorithms revealed that a narrow band S-parameter response carries a significant portion of the information. Therefore, it will be possible to support the open-ended coaxial probes with a narrow band microwave circuitry that is able to collect only S-parameters at low frequencies which decreases the cost of an end product significantly. If realized, such automated microwave diagnostic device can be deployed to many clinics, including rural ones, enabling diagnostic and surgical procedures in remote areas as well as increasing the efficiency of the current protocols in developed regions.

## Methods

### Animals

Sprague Dawley (SD) strain albino female rats was obtained from the Bogazici University, Center for Life Sciences and Technologies, Vivarium division. Animals were randomly divided into control and experiment group. Animals in control group were administered a single dose of pure extra virgin olive oil. Animals in experimental group were administered a single dose 20 mg/kg 7,12-Dimethylbenz(a) anthracene (DMBA) dissolved in 1 ml extra virgin olive oil to initiate mammary carcinoma. Both the dummy solution and the DMBA solution were administered via oral gavage. DMBA was obtained from Sigma Chemical Co in powder form and the target amount was deposited to a colored vial and mixed with 1 ml olive oil. Next, the DMBA was dissolved in olive oil using a closed system ultrasonic bath. Both groups received the solutions at 47 days old since the DMBA must be administered when the animals start reaching puberty. After the administration, the animals were left to recover for 2 weeks. The experiment animal weights were tracked and the animals were checked for tumors by hand every week after 2 weeks rest period ended. The animals had ad libitum access to tap water and standard pellet food during the experiments.

### Experimental setup

**Figure 3:**
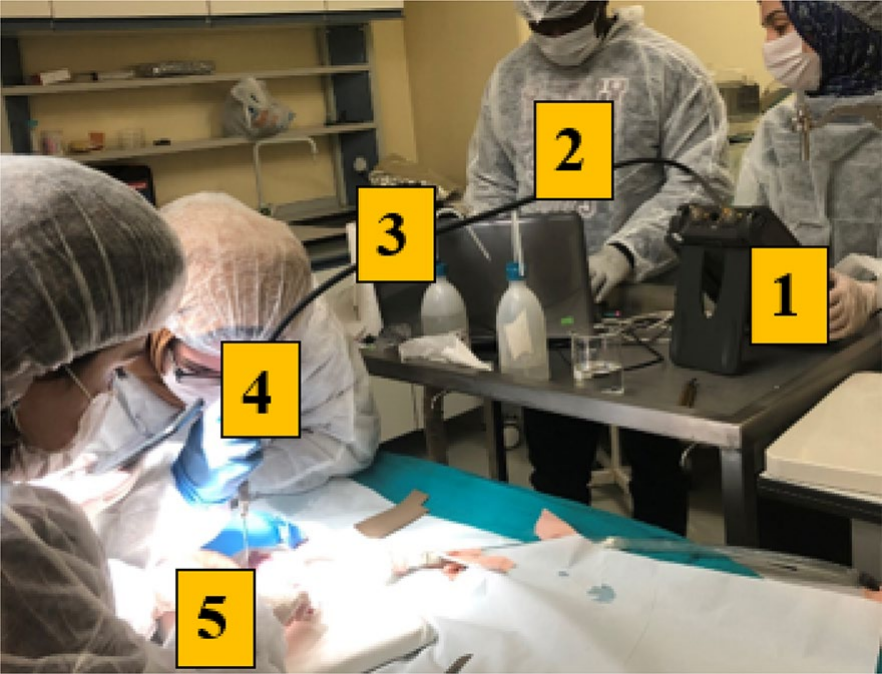
Measurement setup: (**1**) Agilent Fieldfox N9923A Network Analyzer (VNA), (**2**) Laptop computer connected to VNA via local area network (LAN) cable and Agilent IO program, (**3**) Keysight N1501A-202 20 GHz flexible RF cable enables connection between the VNA and the probe, (**4**) Keysight N1501A Dielectric Slim Form Probe, (**5**) rat tissue sample under test.

The dielectric property measurements were performed with the commercially available slim form Keysight N1501A open-ended coaxial probe kit along with Agilent 85070E dielectric measurement software. In addition to the probe kit, the measurement setup, shown in Fig. 3, included the Agilent Fieldfox N9923A Vector Network Analyzer (VNA), a laptop computer, and Keysight N1501A-202 20 GHz flexible RF cable. Please note that the experiments were conducted on animal in vivo tissues and humans shown in Fig. 3 are some of the authors listed in this work. Flexible connection enabled free movement of the probe allowing the replication of the expected cable movements in an in vivo setting. VNA was connected to the computer via Local Area Network (LAN) and Agilent IO program. The S$_{11}$ response was measured by the VNA and recorded while the 85070E software installed in the computer converted the S${_{11}}$ response to the dielectric properties in real time. Both the S${_{11}}$ response and the dielectric properties recorded simultaneously. All measurements were performed between 0.5 and 6 GHz with 0.55 GHz intervals.

In the open-ended coaxial probe technique, the dielectric properties are calculated from the measured reflection coefficient emerging from discontinuity due to the material terminating the probe aperture. This calculation is essentially an ill-posed inverse problem and requires the calculation of network scattering parameters which is done through the calibration procedure. In this work a standard calibration procedure namely open, short and distilled water was employed. To perform the calibration, the open-end (will be referred as aperture throughout this paper) of the probe was simply left in the air for open circuit, next, the aperture was terminated with a conductive textile for short circuit, and lastly, aperture of the probe was immersed in distilled water for a known material load. The temperature of the distilled water was between 21.4 ± 2.5 $^\circ$C.

**Figure 4:**
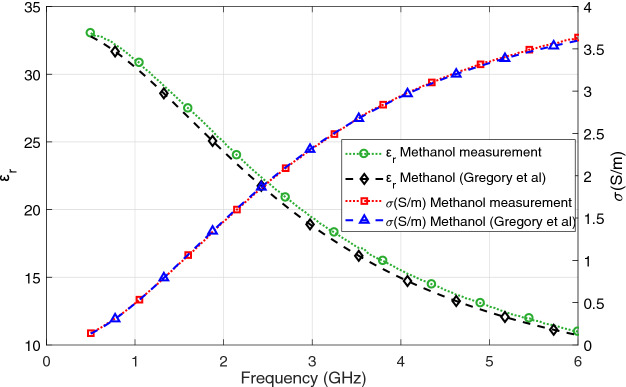
Comparison of measured methanol dielectric properties with the literature data^21^.

Next, calibration validation was performed by collecting measurements from pure methanol, a liquid with well-known dielectric properties. Methanol was preferred due to its relative permittivity, which is in the range of breast tissue also conveniently located between air and distilled water^8^. During measurements, the temperature of the methanol was 21.9 ± 1.9 $^\circ$C. Note that this step was performed before the beginning of the in vivo rat mammary tissue measurements and the calibration was repeated if the methanol measurement differed from the literature data^21^. The mean of the collected 20 pure methanol measurements is compared with literature data (20 $^\circ$C methanol.) Comparison of measurement data with the literature for relative permittivity and conductivity is shown in Fig. 4. From the figure it can be seen that a good agreement is obtained between the literature and measured methanol data. Maximum dielectric property discrepancy between the measured and literature relative permittivity and conductivity are 4.5% at 3.58 GHz and 5.0% at 0.55 GHz, respectively. The obtained results verifies the proper functioning of the measurement setup.

### In vivo measurements

Before the measurements, the probe tip was sterilized using ethyl alcohol (96%) and 2-Propanol mixture. Next, the tip of the probe was washed with distilled water and dried. Then, the standard calibration and verification steps were performed. Again after the verification, the probe tip was washed using distilled water and wiped dry with a cotton pad.

**Figure 5:**
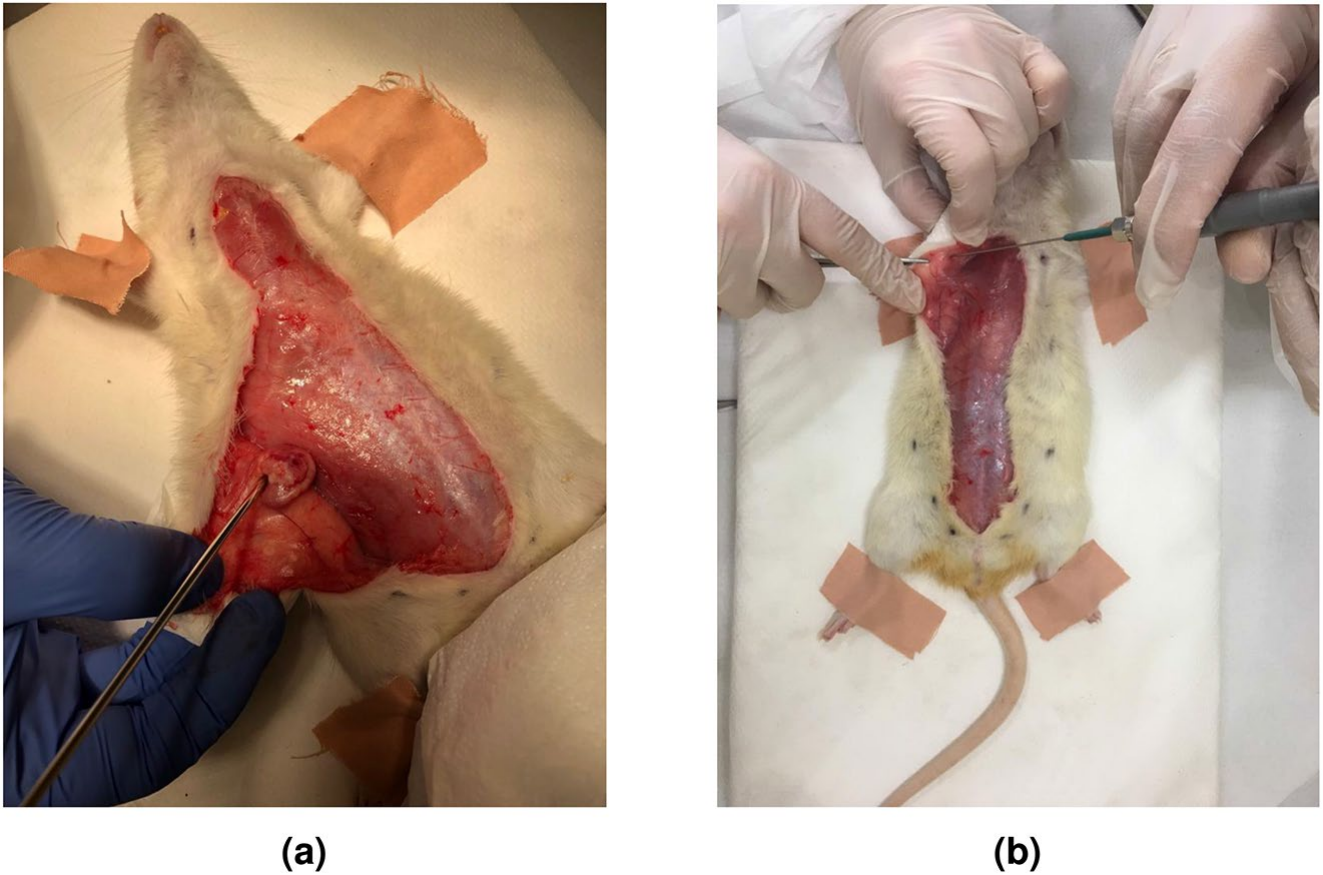
Measurement of samples during in vivo dielectric property and S-parameter response collection: (**a**) rat breast tumor and (**b**) rat breast healthy tissues.

For sample preparation the animals were anesthetized via an intraperitoneal injection of 80mg/kg ketamine and 10mg/kg xylazine mixture. Target biological tissue was accessed via incision and measurements were collected by terminating the probe aperture with the tissue under investigation. Two measurement samples including tumor and healthy breast tissues are shown in Fig. 5a,b, respectively.

Five measurements were taken from each measurement point before moving the probe to another tissue or another point on the same tissue. Before moving the probe to another target measurement point, the probe tip was washed again using distilled water and wiped dry using a cotton pad. Calibration was repeated after every completion of 30 measurements. The calibration and measurements were performed between 0.5 to 6 GHz with 101 frequency points in between.

After the measurements were completed, the measured tissues were excised and placed in an embedding cassette. The cassettes were kept in 10% formalin solution for pathological analysis. Protocols of this experiment were in accordance with the regulations of Bogazici University Institutional Animal Experiments Local Ethics Committee and the research is reported in accordance with the ARRIVE (Animal Research: Reporting of In Vivo Experiments) guidelines. Ethical approval was received from the Bogazici University Institutional Animal Experiments Local Ethics Committee.

### Pathological analysis

**Figure 6:**
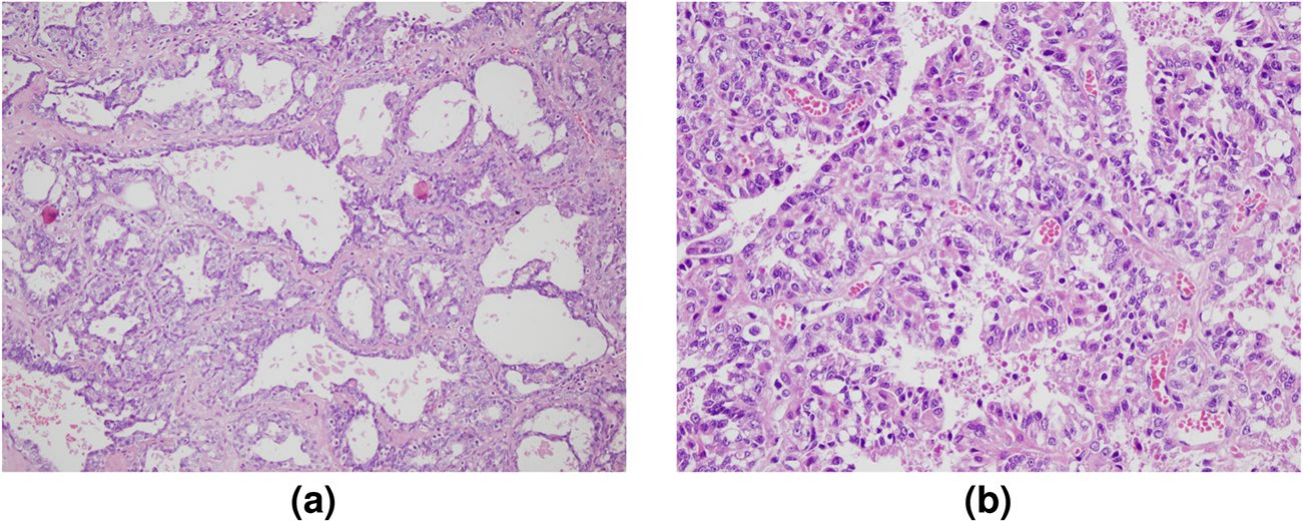
Histopathology picture of samples: (**a**) 10X200 view and (**b**) 15 $\times$ 400 view.

Samples were dehydrated and processed for paraffin embedding, tissue sections were stained with hematoxylin and eosin (HE) for light microscopy studies. The microscope slides were scored based on tubule formation, mitotic activity, and nuclear pleomorphism which is a well established criteria^22^. The microscope slides were examined and scored based on tubule formation, mitotic activity and nuclear pleomorphism. Tubule formation was scored as 1 when more than 75% of tumor shows tubular differentiation, scored as 2 when tubule formation is between 10% to 75%, and scored as 3 when the tumor tubule formation is well under 10%. Mitotic activity was scored by counting the quantity of mitotic figures per 10 high power fields (hbf). Mitoses $\le$7, 8 to 14 and greater than 15 per 10 hpf was scored as 1,2 or 3, respectively. Note that the mitotic count scoring criteria varies depending on the field diameters of the microscopes employed by the pathologist. For the evaluation of nuclear pleomorphism, size and shape variation of the tumor nuclei was taken into account. Uniform cells with small nuclei was scored as 1. Bigger cells and vesicular nuclei, with visible nucleolus was scored as 2. Lastly, marked variation in cells and vesicular nucleus, explicit nucleoli was scored as 3. The overall grade was calculated using total score. The total scores 3-5, 6-7, and 8-9 corresponds to grade 1, 2, and 3, respectively. Samples of pictures of a malignant sample obtained through histopathological analysis with 10X200 and 15X400 view is shown in Fig. 6a and Fig. 6b, respectively.

### Data processing

The measurements were placed on a feature matrix for classification purposes. Between 0.5 and 6 GHz all frequency points were taken as 101 individual complex numbers. Since the real and imaginary parts were considered as separate features, for both dielectric property and S$_{11}$ measurements 202 features were used as input data. To generate a balanced number of measurement samples and to eliminate the bias due to sample size difference 5 animals from each group and 325 measurements from each tissue type were randomly selected. That is, 325 measurements constituting all measurements collected from healthy rat breast tissues were used and 325 measurements were randomly selected from malignant rat breast tumor tissues. To the best of our knowledge, this scale of data have not been presented in the literature before.

### Classification

Classification was performed with the supervised linear classifier using the SVM algorithm^23^. The algorithm is discussed at great length in the literature and many packaged versions are available in various computer languages. The SVM algorithm used in this work was a commercial SVM function in MATLAB named *fitcsvm*. Briefly, SVM works by constructing a hyperplane to separate the multi dimensional data. To do so, the data points close to the boundary between two classes are selected and named support vectors. The goal is to find a hyperplane that will have a maximum distance, called margin, from the support vectors. SVM is implemented using kernel functions. These functions are mathematical operators that transforms the input data to desired form that can potentially aid constructing a hyperplane that can separate the input data with targeted accuracy. In this work, linear and RBF kernel functions were used. All data processing and classification described in this work was performed with MATLAB language.

**Figure 7:**
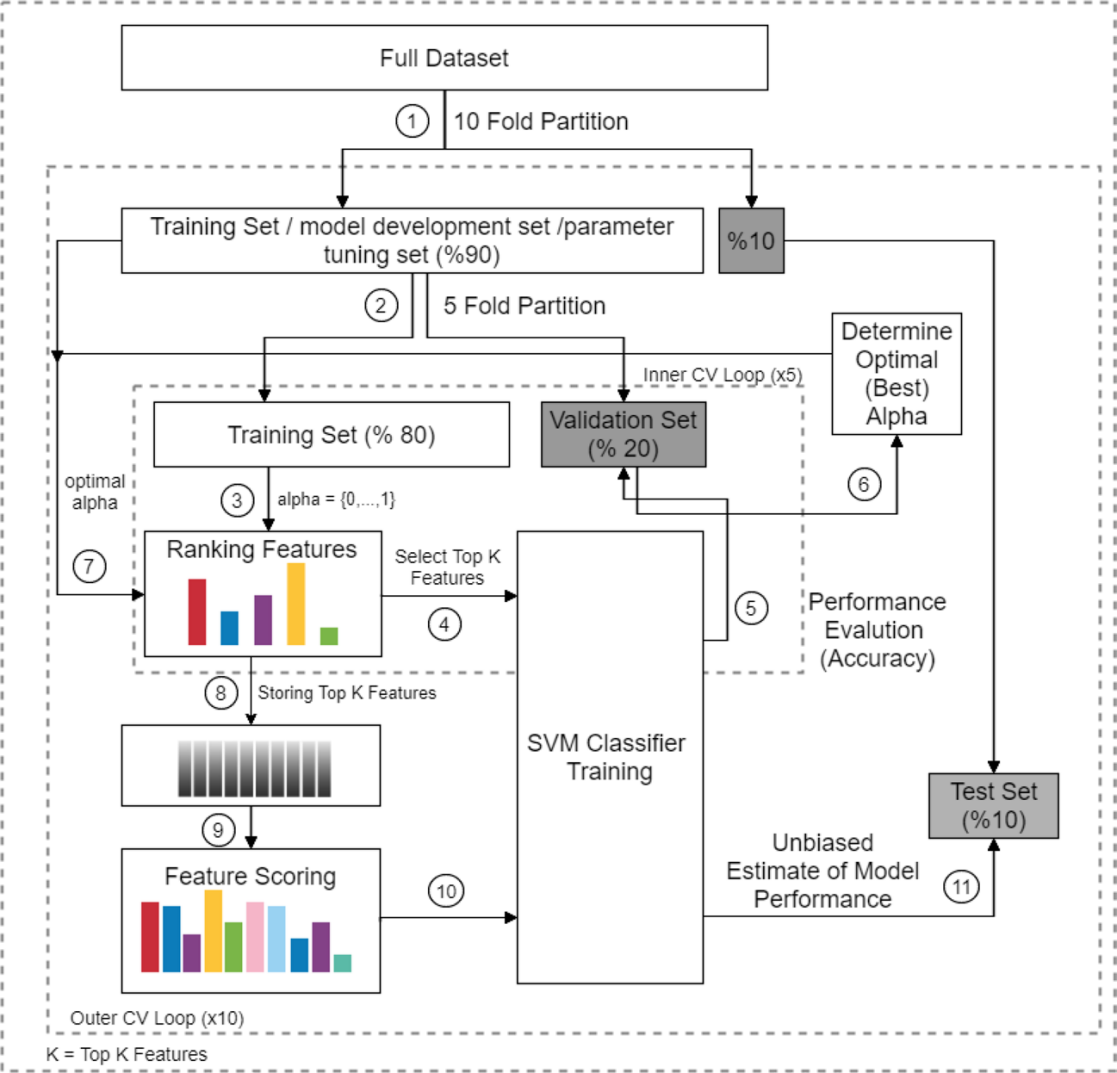
Flow chart for nested cross validation (CV) scheme.

### Feature selection and reproducibility using multiple cross-validation schemes

Feature selection (FS) was only applied to S$_{11}$ response. The purpose of applying FS to the S$_{11}$ response is to investigate the potential of narrow band applications. In this study, infinite feature selection (Inf-FS)^24,25^ along with the SVM classification algorithm were applied to extract the most discriminative feature. Since Inf-FS is proven to outperform other feature selection (FS) methods on multiple benchmark data sets, we implemented the Inf-FS algorithm in this work. Being one of the filter class FS methods, Inf-FS operates by mapping the FS problem to an affinity diagram that is built with nodes and paths corresponding to features and subset of features, respectively. The goal is to rank the features by calculating an energy score. The energy score is assigned by evaluating the combination of path length and pairwise energy term. Pairwise energy term is a weighted combination of standard deviation over samples and a correlation coefficient. The weight of each term is determined via loading coefficient, denoted as $\alpha$ ranges between [0,1], and it was optimized for each scenario via nested Cross Validation (CV). The Inf-FS returns a rank and a weight matrix. In this work, the returned rank matrix was stored for each CV step and the features were scored based on the frequency and rank order. Desired SVM algorithm is then trained and tested with the predetermined K number of features with 10 fold CV scheme.

**Figure 8:**
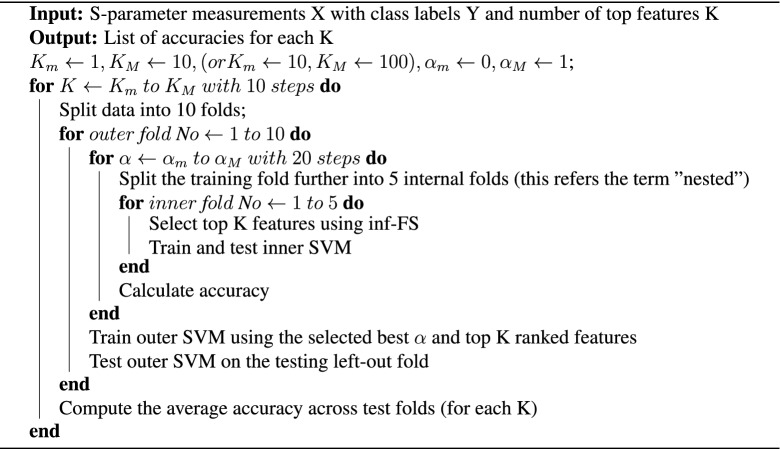
Algorithm of nested cross validation (CV).

Fig. 7 shows a flowchart of the nested CV algorithm used in this work. The algorithm is also described with a pseudocode as well in Fig. 8. Briefly, the dataset was splitted into 10 folds. One fold was reserved for validation and the remaining data were used at the outer CV loop for training. Note that, parameter $\alpha$ was tuned during training. The $\alpha$ varied between [0,1] with 0.05 intervals and the optimal $\alpha$ was found through training by calculating the accuracy for each $\alpha$ value. To do so, the training fold was further divided into 5 internal folds, referred as “nested”. At each inner CV loop, one of fold was used as a validation set. Other folds constituted inner training set on which inf-FS was applied. Next, top K features was selected and SVM algorithm was trained. Accuracy was calculated using test set. First the optimal $\alpha$ parameter with maximum accuracy was determined for each outer loop and inf-FS was applied using the optimal $\alpha$.

## Supplementary Information

Supplementary Information 1.

Supplementary Information 2.

Supplementary Information 3.

## References

[1] Gabriel, S., Lau, R. & Gabriel, C. The dielectric properties of biological tissues: Ii. measurements in the frequency range 10 hz to 20 ghz. *Phys. Med. Biol.* **41**, 2251 (1996). 10.1088/0031-9155/41/11/002 8938025

[2] Lazebnik M (2007). A large-scale study of the ultrawideband microwave dielectric properties of normal, benign and malignant breast tissues obtained from cancer surgeries. Phys. Med. Biol..

[3] Tunçay AH, Akduman I (2014). Realistic microwave breast models through t1-weighted 3-d mri data. IEEE Trans. Biomed. Eng..

[4] Converse M, Bond EJ, Veen B, Hagness C (2006). A computational study of ultra-wideband versus narrowband microwave hyperthermia for breast cancer treatment. IEEE Trans. Microw. Theory Tech..

[5] Cendán JC, Coco D, Copeland EM (2005). Accuracy of intraoperative frozen-section analysis of breast cancer lumpectomy-bed margins. J. Am. Coll. Surg..

[6] Levine H, Papas CH (1951). Theory of the circular diffraction antenna. J. Appl. Phys..

[7] Anderson, W. Microwave biopsy probe (2006). US Patent App. 10/961,812.

[8] Popovic D (2005). Precision open-ended coaxial probes for in vivo and ex vivo dielectric spectroscopy of biological tissues at microwave frequencies. IEEE Trans. Microw. Theory Tech..

[9] Gabriel C, Chan T, Grant E (1994). Admittance models for open ended coaxial probes and their place in dielectric spectroscopy. Phys. Med. Biol..

[10] Maenhout G, Markovic T, Ocket I, Nauwelaers B (2020). Effect of open-ended coaxial probe-to-tissue contact pressure on dielectric measurements. Sensors.

[11] La Gioia A (2018). Open-ended coaxial probe technique for dielectric measurement of biological tissues: Challenges and common practices. Diagnostics.

[12] Cho J (2006). In-vivo measurements of the dielectric properties of breast carcinoma xenografted on nude mice. Int. J. Cancer.

[13] Kim T-H, Pack J-K (2012). Measurement of electrical characteristics of female breast tissues for the development of the breast cancer detector. Progr. Electromagn. Res. C.

[14] Martellosio, A. *et al.* Dielectric properties characterization from 0.5 to 50 ghz of breast cancer tissues. *IEEE Trans. Microw. Theory Tech.* **65**, 998–1011 (2016).

[15] Cheng Y, Fu M (2018). Dielectric properties for non-invasive detection of normal, benign, and malignant breast tissues using microwave theories. Thoracic Cancer.

[16] Lin J (1985). Frequency optimization for microwave imaging of biological tissues. Proc. IEEE.

[17] Shen, X. *et al.* Using connectome-based predictive modeling to predict individual behavior from brain connectivity. *Nat. Protoc.* **12**, 506 (2017). 10.1038/nprot.2016.178 PMC5526681 28182017

[18] Georges, N. *et al.* Identifying the best data-driven feature selection method for boosting reproducibility in classification tasks. *Pattern Recogn.* **101**, 107183 (2020).

[19] Saçlı, B. *et al.* Microwave dielectric property based classification of renal calculi: Application of a knn algorithm. *Comput. Biol. Med.* **112**, 103366 (2019). 10.1016/j.compbiomed.2019.103366 31386972

[20] Yilmaz T (2016). Machine learning aided diagnosis of hepatic malignancies through in vivo dielectric measurements with microwaves. Phys. Med. Biol..

[21] Gregory, A. P. & Clarke, R. Tables of the complex permittivity of dielectric reference liquids at frequencies up to 5 ghz. *NPL Report MAT 23* (2012).

[22] Kim MS (2011). Oral administration of loquat suppresses dmba-induced breast cancer in rats. Food Sci. Biotechnol..

[23] Andrew, A.M. An introduction to support vector machines and other kernel-based learning methods by Nello Christianini and John Shawe-Taylor, Cambridge University Press, Cambridge, 2000, xiii+ 189 pp., ISBN 0-521-78019-5 (hbk,£ 27.50). *Robotica* **18**, 687–689 (2000).

[24] Roffo, G., Melzi, S. & Cristani, M. Infinite feature selection. In *Proceedings of the IEEE International Conference on Computer Vision*, 4202–4210 (2015).

[25] Adeli E (2017). Kernel-based joint feature selection and max-margin classification for early diagnosis of Parkinson’s disease. Sci. Rep..

