# Supplementary material for: Classification of rat mammary carcinoma with large scale in vivo microwave measurements
Source: Sci Rep. 2022 Jan 10;12:349. doi: 10.1038/s41598-021-03884-7 (PMC8748494; doi:10.1038/s41598-021-03884-7)

Description of the dataset

This dataset includes two files from Microwave measurements of rat heathy breast tissues and breast tumors obtained using open-ended coaxial probe. Data was collected between 0.5 and 6 GHz with equally spaced 101 frequency points.

Each file contains 650 samples corresponding to 325 healthy and 325 tumor measurements.

Dielectric property data can be found in "data_perm_cond.xlsx"; 202 features corresponding to 101 relative permittivity (first 101 features) and 101 conductivity values (last 101 features).

S-parameters data can be found in "data_s_parameters.xlsx"; total 202 features including real part (first 101 features) and imaginary part (last 101 features).

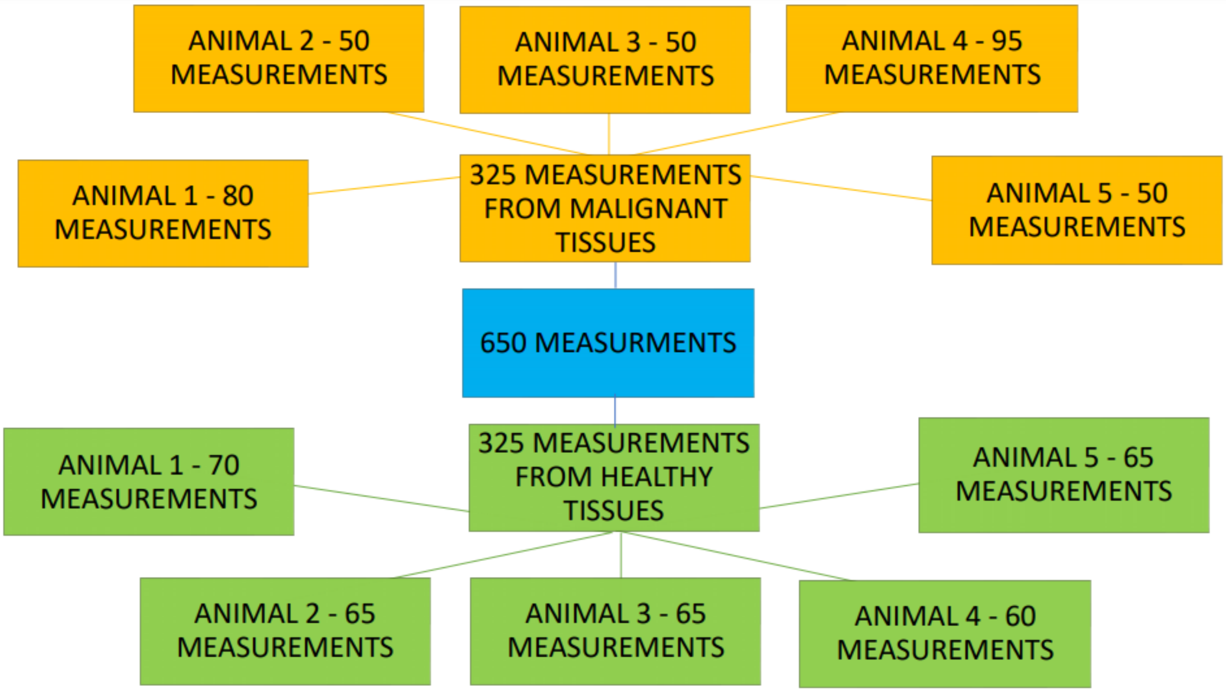

Supplement: Supplementary file 3 — Supplementary Information 3. [file 41598_2021_3884_MOESM3_ESM.pdf]
